# Alpha modulation via transcranial alternating current stimulation in adults with attention-deficit hyperactivity disorder

**DOI:** 10.3389/fpsyg.2023.1280397

**Published:** 2024-01-12

**Authors:** Kyra Kannen, Johanna Rasbach, Amin Fantazi, Annika Wiebe, Benjamin Selaskowski, Laura Asché, Behrem Aslan, Silke Lux, Christoph S. Herrmann, Alexandra Philipsen, Niclas Braun

**Affiliations:** ^1^Department of Psychiatry and Psychotherapy, University Hospital Bonn, Bonn, Germany; ^2^Experimental Psychology Lab, Department of Psychology, University of Oldenburg, Oldenburg, Germany

**Keywords:** attention, ADHD, alpha, virtual reality, brain stimulation, tACS

## Abstract

**Background:**

One potential therapy treating attention-deficit/hyperactivity disorder (ADHD) is to modulate dysfunctional brain activations using brain stimulation techniques. While the number of studies investigating the effect of transcranial direct current stimulation on ADHD symptoms continues to increase, transcranial alternating current stimulation (tACS) is poorly examined. Previous studies reported impaired alpha brain oscillation (8–12 Hz) that may be associated with increased attention deficits in ADHD. Our aim was to enhance alpha power in adult ADHD patients via tACS, using different methods to explore potential therapeutic effects.

**Methods:**

Undergoing a crossover design, adults with ADHD received active and sham stimulation on distinct days. Before and after each intervention, mean alpha power, attention performance, subjective symptom ratings, as well as head and gaze movement were examined.

**Results:**

Frequency analyses revealed a significant power increase in the alpha band after both interventions. Despite a trend toward an interaction effect, this alpha power increase was, however, not significantly higher after active stimulation compared to sham stimulation. For the other measures, some additional pre-post effects were found, which were not intervention-related.

**Conclusion:**

Our study cannot provide clear evidence for a tACS-induced increase in alpha power in adult ADHD patients, and thus no stimulation related improvement of attention parameters. We provide further recommendations for the future investigation of tACS as a potential ADHD treatment.

## 1 Introduction

To alleviate their inattention, hyperactivity and impulsivity, adults with attention-deficit/hyperactivity disorder (ADHD) often receive long-lasting psychopharmacological treatment. While this form of treatment is still yielding the greatest success for adult ADHD, it can be accompanied by undesirable side effects, such as weight loss and sleep disturbances (Graham et al., 2011; Wynchank et al., 2017; Kis et al., 2020). In addition, psychostimulants appear to be less effective in adult ADHD patients than in children and adolescents with ADHD (Wilens et al., 2011; Cortese et al., 2018). Although ADHD medication has shown high short-term efficacy in many studies (Mészáros et al., 2009; Cunill et al., 2016), their longer-term efficacy awaits further investigation (Cortese et al., 2018; Swanson, 2019) given that several patients seem to develop tolerance to psychostimulants (Handelman and Sumiya, 2022).

In view of these drawbacks of psychopharmacological ADHD treatment, in the last decade various potential alternatives to non-pharmacological treatment have been investigated that enable ADHD treatment without or with fewer side effects. Besides psychotherapeutic approaches, for instance, physical activity training (Barudin-Carreiro et al., 2022; Montalva-Valenzuela et al., 2022; Seiffer et al., 2022), herbal treatments (Sarris et al., 2011), and digital health interventions (Lakes et al., 2022), including virtual reality (VR) interventions (for review, see Bashiri et al., 2017; Romero-Ayuso et al., 2021) and app-based psychoeducation (Selaskowski et al., 2022, 2023b) have been investigated. The probably most famous and controversially discussed alternative ADHD treatment approach, however, is still neurofeedback. This therapy intervention aims to improve the self-regulation of brain activity and has been under investigation for almost 50 years (Arns et al., 2014). While some researchers conclude positive effects of neurofeedback on ADHD symptoms (see, e.g., systematic review by Moreno-García et al., 2022) others have been more sceptical (for a systematic review and meta-analysis, see Louthrenoo et al., 2022; Rahmani et al., 2022). Therefore, its efficacy remains unclear. Accordingly, there is still a substantial need for developing more effective ADHD treatment approaches with less side effects.

Another treatment approach, though still in its infancy, is the idea of using brain stimulation techniques in place of, or as an adjunct to, traditional treatments. So far, the most established non-invasive brain stimulation techniques are transcranial magnetic stimulation (TMS) and transcranial electrical stimulation (TES). While TMS is delivered by a pulsing electromagnetic coil that is held next to the skull, in TES, multiple electrodes are placed onto the scalp to apply an electrical current to decrease or increase neural activity (Vosskuhl et al., 2018). Prominent TES subtypes are transcranial direct current stimulation (tDCS) and transcranial alternating current stimulation (tACS). While under tDCS a constant current is applied, under tACS the current alternates at a specified frequency (Herrmann et al., 2013). Accordingly, the respective mechanism of action on brain activity is different: Whereas tDCS seeks to increase or decrease the general neuronal excitability in a stimulated brain area of interest depending on the type of stimulation used, tACS seeks to amplify a specific brain oscillation by stimulating the brain with the dominant frequency of the oscillation of interest. Notably, both methods are thereby considered safe and with few side effects (Vosskuhl et al., 2018; Westwood et al., 2021).

Although various studies have already investigated TMS and tDCS as possible treatment approaches for ADHD (for systematic reviews, see Salehinejad et al., 2020; Westwood et al., 2021; Chen et al., 2023), only few clinical investigations addressed the efficacy and tolerability of tACS for ADHD treatment. In fact, to our knowledge, only three studies have so far explored tACS as treatment for adult ADHD (Dallmer-Zerbe et al., 2020; Farokhzadi et al., 2020; Kannen et al., 2022). While one of the studies compared tACS to methylphenidate (Farokhzadi et al., 2020) and reported tACS as an effective treatment, the other two studies investigated tACS as an alternative treatment for ADHD by trying to increase the P300 amplitude (Dallmer-Zerbe et al., 2020; Kannen et al., 2022), which is considered to be diminished in ADHD patients (Hasler et al., 2016; Marquardt et al., 2018; Kaiser et al., 2020). Dallmer-Zerbe et al. (2020) observed an increase in the P300 amplitude accompanied by a decrease in omission errors among adult ADHD patients, whereas Kannen et al. (2022) did not confirm these results. Therefore, the extent to which tACS might be beneficial in treating ADHD remains unclear.

Besides the diminished P300, another possible neuronal target for the application of tACS could be the brain’s alpha rhythm (8–12 Hz), which is known to be modulated during attention and considered as a potential biomarker for ADHD (Kiiski et al., 2020). In healthy individuals, alpha oscillations are dominant in posterior brain regions during relaxed wakefulness, and progressively relocate towards central and frontal cortical regions with increasing drowsiness (see, e.g., Goldman et al., 2002). The hypothesis thereby is that alpha oscillations enable basal cognitive functions and attentional processes (Klimesch, 2012). Moreover, of particular interest in the present context, alpha oscillations are reported to be reduced in ADHD patients in both power and frequency (Loo et al., 2009; Woltering et al., 2012; Poil et al., 2014; Liu et al., 2016; Deiber et al., 2020), although this finding could not be corroborated in other studies (for discussion, see Adamou et al., 2020). In addition, in line with this assumed alpha alleviation, some studies showed that increasing alpha power using neurofeedback resulted in clinical improvement of ADHD symptoms as well as in an increase of attentional performance (Bazanova et al., 2018; Deiber et al., 2020). Considering these findings, the question arises whether a tACS-induced increase of the participant’s individual alpha activity might improve the attentional performance of ADHD patients.

To prove a tACS-induced improvement of impairments in attentional functions, however, the difficulty arises that such ADHD symptoms often cannot be reliably detected with standard neuropsychological tests. One potential factor for this limited diagnostic utility might be the low ecological validity, which might fail to mimic everyday life challenges of ADHD patients (Wasserman and Wasserman, 2012; Varao-Sousa et al., 2018). A possible solution for creating more reality-close test situations might be offered by VR technology. By creating three-dimensional, immersive, and interactive virtual environments which allow to mimic everyday life demands, ecological validity can be increased while maintaining a high level of standardization (Parsons, 2015).

The aim of the present study was to increase the individual alpha power in patients with adult ADHD and to investigate possible behavioral and neurophysiological changes resulting therefrom. To this end, a crossover trial was carried out, in which all patients underwent both an individual tACS-based alpha stimulation (active stimulation) and a placebo stimulation (sham stimulation). To simulate an everyday situation, a developed virtual seminar room (VSR) was used that allowed for a multimodal and standardized, but symptom-valid measurement of inattention, hyperactivity and impulsivity (Wiebe et al., 2022, 2023; Selaskowski et al., 2023a).

## 2 Materials and methods

### 2.1 Participants

Twenty-seven ADHD patients volunteered in this study, out of which 24 (7 female; *M_age_* = 32.25, *SD_age_* = 10.46, aged between 19 and 53) completed the experiment. The recruitment of the sample was conducted via the specialized outpatient clinic for adult ADHD of the Department of Psychiatry and Psychotherapy at the University Hospital Bonn. Participants were either personally invited to the study during medical consultations or via a study applicant pool in which they had registered before. The study was approved by the medical ethics committee of the University of Bonn (protocol number: 195/20), conducted in accordance with the Declaration of Helsinki, and pre-registered at the German Clinical Trials Register (https://www.drks.de/, Trial-ID: DRKS00022927). Written informed consent was obtained from all participants and they all received a monetary compensation of 25 € for their participation.

### 2.2 Study design and general procedure

The trial was carried out as a crossover study with two interventions on three measurement days: “active stimulation” (the true tACS intervention) and “sham stimulation” (the placebo intervention). On Day 1, a comprehensive clinical examination was performed during which the ADHD diagnosis was validated, and comorbidities were evaluated. On Days 2 and 3, the stimulation experiment took place, with one of the two interventions being applied on each measurement day. The order of interventions (sham stimulation or active stimulation) was counterbalanced.

### 2.3 Eligibility assessment and clinical characterization

For confirmation of the ADHD diagnoses and further characterization of the individual ADHD symptom profiles, all participants were administered the structured clinical “Interview of Integrated Diagnosis of ADHD in Adulthood” (IDA-R; Retz et al., 2014). In addition, to check for exclusion criteria and to assess potential comorbidities, the German version of the “Diagnostic Short Interview for Mental Disorders” (Mini-Dips-OA; Margraf et al., 2017) was carried out. Both clinical interviews were conducted via video call using the online-platform RED medical.^1^ Moreover, participants completed a battery of online-surveys, including, for instance, a demographic questionnaire, a questionnaire concerning quality of life (WHO-QOL; Harper et al., 1998) and the ADHD Self-Report-Scale (ADHS-SB; Rösler et al., 2004).

To be eligible for the study, participants needed to be right-handed (according to the Edinburgh Handedness Inventory; Oldfield, 1971), to be between 18 and 60 years old, and to have corrected-to-normal or normal vision. In addition, any of the following exclusion criteria had to be absent: current severe major depression or current substance dependence, psychosis, presence of a serious neurological disorder (especially epilepsy), presence of a dermatological disorder of the head, pregnancy, or no command of the German language. Intake of ADHD medication (reported by 12 participants of the final cohort) was discontinued 24 h prior to each of the laboratory sessions. Participants were instructed to abstain from caffeine and alcohol for at least 24 h before each laboratory session.

### 2.4 Experimental procedure

The experiment took place in the VR laboratory of the Department of Psychiatry and Psychotherapy at the University Hospital in Bonn and was scheduled at two separate appointments. On one appointment the active stimulation was applied, while on the other appointment only a sham stimulation was applied. Each appointment started with the preparation of tACS- and EEG-electrodes. Afterwards, participants took their seat in front of a 1 × 1 m table within a 3.70 m x 2.65 m VR play area. The active experiment started by measuring 2 min of resting state baseline EEG, followed by the determination of the individual alpha frequency (IAF). Once the IAF was determined, participants became equipped with the head mounted display *HTC Vive Pro Eye* (HTC Corporation, Taoyuan City, Taiwan) and entered the VSR. Immersed into the VSR, participants were familiarized with this new virtual environment as well as with the continuous performance task (CPT) that next would take place within the VSR (*cf.* section 2.5). In total, three CPT blocks were presented, whereby each CPT block lasted 18 min and was suspended by a two-minute resting state EEG measurement and a one-minute-long break. Moreover, after each block, the participants’ subjective ADHD symptoms (one question regarding inattention, impulsivity, hyperactivity, respectively, answered on a 7-point Likert-scale) was prompted by a gesture-controlled user interface inside VR (for further details, see Wiebe et al., 2022). Finally, after the last CPT block ended, participants completed the Virtual Reality Sickness Questionnaire (VSRQ; Kim et al., 2018) and a questionnaire about tACS side effects (Brunoni et al., 2011). Also, to investigate if participants were blinded to the experimental condition, they were asked whether they thought they received the active stimulation or sham stimulation.

### 2.5 Virtual seminar room and continuous performance task

**Figure 1:**
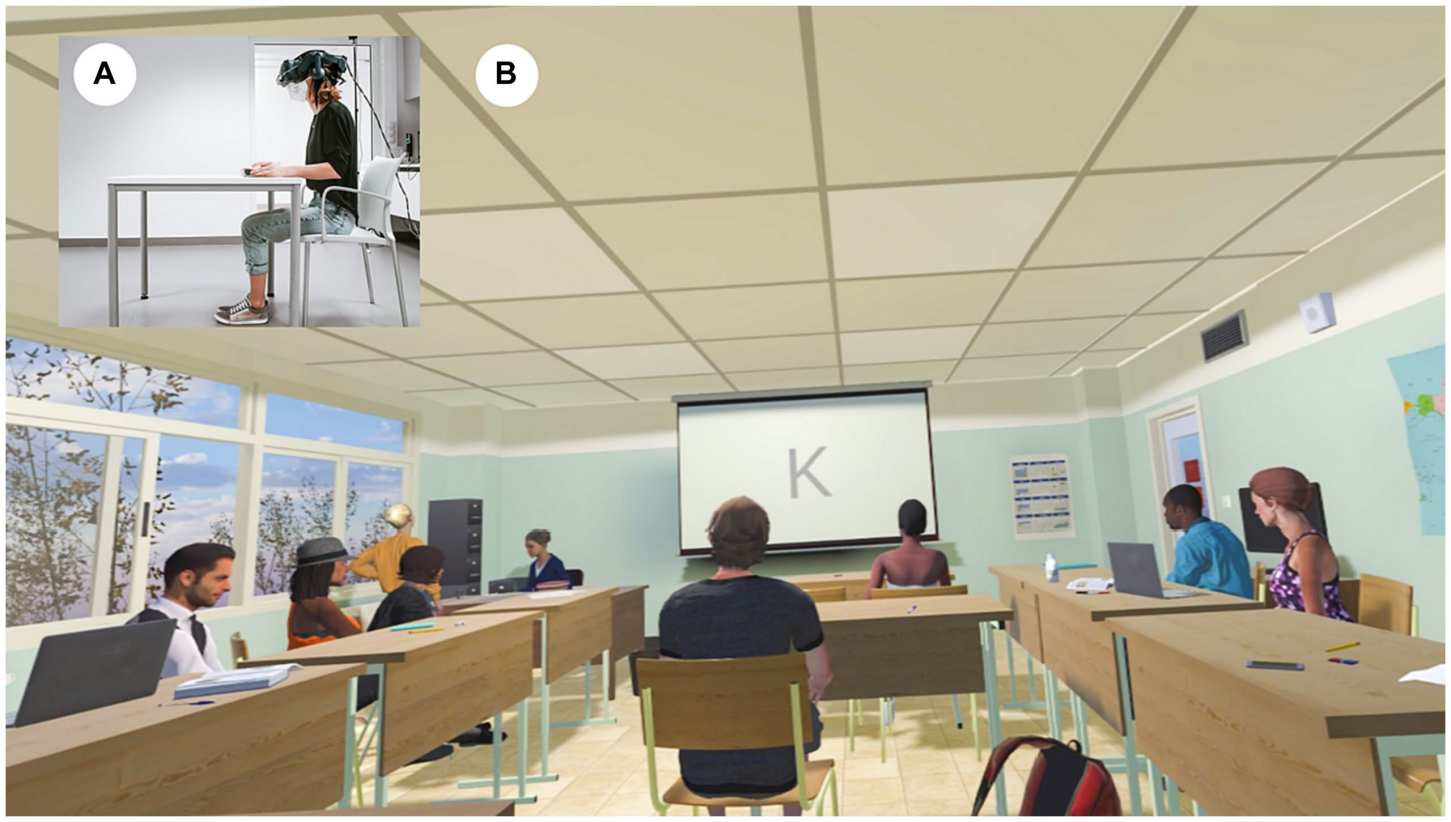
The virtual seminar room (VSR). **(A)** Real-world third-person perspective and **(B)** virtual-environment first person perspective. Adults with ADHD were immersed into the VSR, in which the continuous performance task (CPT) was presented at the canvas. **(A)** is an exemplary depiction and thus without attached tACS. For programming the virtual seminar room we only used non-restricted assets. “School Classroom” (Reprinted from 3D Everything via Unity Asset Store, licensed under Standard Unity Asset Store EULA).

The VSR and the implemented CPT are depicted in Figure 1 and have been described in detail previously (Wiebe et al., 2022). In brief, based on existing assets (i.a. the “School Classroom” from 3D everything available in the Unity Asset Store), the VSR was developed under Unity 3D version 2019.1.10f1 (Unity technologies, San Francisco, CA, United Staes) and contained the typical furniture found in a seminar room, including chairs and tables as well as a canvas at the front of the VSR. Moreover, the VSR comprised virtual classmates that performed unobtrusive idle movements during non-distractor phases (NDP) and more complex actions during distractor phases (DP; details below). The virtual table where the participants found themselves seated, was thereby located in the back of the VSR, so that participants had a good overview of the entire VSR.

The CPT itself was presented on the canvas and consisted of a pseudorandomly-presented series of letters ranging from “A” to “Z”, each presented with a 1.1 s inter-stimulus-interval and 100 ms duration. The task was to press the space bar as soon as the letter “A” was followed by the letter “K”, while in all other cases, a response had to be withhold (Neguț et al., 2017; Mühlberger et al., 2020). After a practice run of 20 trials, the actual CPT began, which was split into three consecutive blocks: A pre-intervention block that occurred before active or sham stimulation was applied; a during-intervention block in which the active or sham stimulation was applied; and a post-intervention block that occurred after the active or sham stimulation.

Each of the CPT-blocks thereby lasted approximately 18 min and included 450 letter pairs, partitioned into 135 target sequences (~30%) and 315 non-target sequences (~70%). To elevate task difficulty, non-target sequences included 158 pseudo target sequences (“K” not preceded by “A”). Furthermore, each CPT block consisted of three DP and three NDP, each lasting three min. While during NDP no distractors were played, during DP, 54 different distracting events were played in total, of which 18 were exclusively visual (e.g., a paper airplane), another 18 solely auditory (e.g., a bell noise) and the remaining 18 audiovisual (e.g., passing fire trucks). Across participants, the order of distractors was thereby randomized, and the order of phases counterbalanced.

For analyzing CPT-performances, three main parameters of interest were defined: Omission error rate (i.e., the percentage of missed responses to target stimuli), commission error rate (i.e., the percentage of invalid responses to non-target stimuli) and reaction time variability (RTV, i.e., the standard deviation of reaction times towards correct hit trials divided by the mean reaction time). While omission error rates are regarded to reflect inattention, commission error rates are considered to reflect impulsive behavior (Nichols and Waschbusch, 2004), and RTV is considered a measure of vigilance (Llevy et al., 2018).

### 2.6 Electrical brain stimulation and electrode montage

The tACS was delivered by a battery-operated stimulator system (DC Stimulator Plus, Neuroconn, Illmenau, Germany). With the help of an electrically conductive paste (ten20 conductive paste, Weaver and Co., Aurora, CO, United States), two rubber electrodes were attached to the participants’ scalp. Since former studies reported significant differences in the alpha band power of posterior brain regions between ADHD patients and healthy controls (see scalp plots, e.g., Woltering et al., 2012; Deiber et al., 2020), one electrode was placed above Cz (5 × 7 cm) and another above Oz (4 × 4 cm). Modeling studies have shown that this montage achieves the highest current densities in posterior brain regions (Neuling et al., 2012) and elicits aftereffects in alpha band power (Neuling et al., 2013; Kasten et al., 2016). Impedances were kept below 15 kΩ (*M* = 4.55, *SD* = 2.92). Participants were stimulated at their IAF (9.63 Hz ± 0.69 Hz active stimulation, 9.67 Hz ± 0.98 Hz sham stimulation) with an intensity of 1.5 mA. Baseline resting-EEG measurements (2 min, eyes open) for determining the IAF were performed before the actual experiment and outside VR (for analysis steps *cf.* section 2.4.1). After the first CPT block, participants received either 18 min of tACS (active stimulation) or 10 s of tACS (sham stimulation) with 10 s fade-in and fade-out (30 s in total to evoke a light tingling sensation in both conditions, implemented for blinding purposes). This sham stimulation procedure is one of the commonly used placebo stimulation techniques (Davis et al., 2013).

### 2.7 EEG recording and analysis

To acquire electroencephalography (EEG) data, we used a wireless EEG system (Smarting^®^, mBrainTrain^®^, Belgrade, Serbia) with 22 Ag/AgCl sintered ring electrodes (Fp1, Fp2, AFz, F3, Fz, F4, T7, C3, Cz, C4, T8, CPz, P7, P3, Pz, P4, P8, POz, O1, O2, M1, M2) of the international 10/20 system that were mounted to an elastic EEG cap (Easycap, Herrsching, Germany). While electrode FPz served as ground, FCz served as reference electrode. The amplifier was connected via Bluetooth with the recording computer. Data was sampled at 500 Hz frequency via Lab Streaming Layer (LSL)^2^ and all impedances were kept below 15 kΩ. EEG data were processed with Matlab 2021b (MathWorks Inc., Natick, MA, United States), using EEGLAB 2021.0 (Delorme and Makeig, 2004) and in-house scripts.

#### 2.7.1 On-site analysis of IAF

For the evaluation of the individual stimulation frequency, resting EEG at channel Pz was filtered between 0.1 and 40 Hz and epoched into 2 s long segments. Afterwards, non-stereotyped artifacts were removed using built-in EEGLAB functions (joint probability test, ±1.7-SD single-channel and global-channel thresholds) before an independent-component-analysis (ICA) (“fastica” version) was conducted. After visual inspection of the generated ICA components, artifacts like vertical and horizontal eye movements were identified and removed in the continuous EEG data set. Clean continuous EEG data from channel Pz was epoched into 2 s long segments and the frequency power spectrum was extracted by Matlab’s *pspectrum()* function between 0 and 40 Hz. The resulting frequency resolution was 0.05 Hz, while the resulting time resolution amounted to 0.25 s. Next, the power spectra were logarithmized and averaged across trials. Finally, the maximum alpha frequency between 7 and 13 Hz was used for the calculation of stimulation parameters.

#### 2.7.2 Stereotyped artifact removal for offline wavelet analysis

Before wavelet analyses were performed, the EEG datasets were cleaned from stereotypic artifacts by the following steps: First, the EEG data was resampled to 250 Hz, filtered between 1 and 40 Hz, and detrended. Second, due to tACS artifacts during stimulation, the second CPT block was removed. Third, noisy EEG channels were detected (6 datasets, *M* = 1.67, *SD* = 0.82) and replaced via spherical interpolation. Fourth, for computing an independent component analysis (ICA), the continuous EEG data was segmented into 2 s time windows and non-stereotypic artifacts were removed using built-in EEGLAB functions (joint probability test, ± 2-SD single-channel and global-channel thresholds). Fifth, the ICA (“extended” version) was computed on the epoched data and components reflecting horizontal or vertical eye movements, heartbeat, muscle activity, or electrode artifacts were visually identified, backprojected to the continuous EEG data, and then rejected. All components that included a 10 Hz peak were retained.

#### 2.7.3 Offline wavelet analysis of alpha activity during CPT blocks

One wavelet analysis focused on potential differences in alpha activity between blocks (pre-intervention vs. post-intervention block) and interventions (active stimulation vs. sham stimulation) during CPT performance. To this end, the ICA-corrected continuous EEG datasets were split into four segmented subsets, such that each subset represented one of the four compared conditions and entailed as many non-overlapping 2 s EEG segments as available within the CPT block of the respecting condition. Next, the following identical pre-processing and analysis steps were performed on each subset: First, the same non-stereotypic artifact removal was conducted that had already been conducted for the ICA calculation. Second, additional non-stereotypic artifact removal was conducted with the help of an eeglab plugin (Ben-Shachar, 2020), in that within each epoch, channels that exceeded 150 μV were marked for rejection. If the channels being marked for rejection were noisy in more than 15% of all epochs, the channels were excluded. In addition, epochs with more than 10 identified bad channels were rejected, while epochs with less than 10 bad channels were included, whereby bad-channel data was replaced by spherical interpolation. Third, a continuous wavelet transformation (CWT) was calculated on each retained epoch of the respective dataset (intervention) for channels Pz, POz, CPz, P3, P4. The frequency range obtained thereby reached from 0.27 Hz to 30.00 Hz in 69 steps on a log scale and the time resolution amounted to 0.004 s. After that, the derived power spectra were logarithmized and a mean power spectrum was derived by averaging across all derived power spectra. Finally, for statistical analyses, the mean alpha power (7–13 Hz) across all five channels for both blocks (pre intervention/post intervention) and both interventions (active stimulation/sham stimulation) was derived by taking the average power across all frequency bins falling into the respecting frequency range and time range between 0.2 and 1.8 s. To check for outliers, the pre-to-post-difference for alpha power was calculated and it was examined whether any datasets differed ±2 standard deviations from the mean alpha power change.

#### 2.7.4 Offline wavelet analysis of alpha activity during resting states

Another wavelet analysis focused on potential differences in alpha activity between blocks and interventions during the 2 min resting state phases. Here, the preprocessing steps were identical to the just described wavelet analysis on the CPT blocks, with the only exception that the segmentation into the four individual subsets was not based on the CPT blocks themselves, but on the 2 min resting state phases. The obtained frequency range and time range was the same as reported above (*cf.* section 2.4.3).

#### 2.7.5 Eye tracking recording and analyses

Eye tracking analyses focused on differences in gaze behavior between blocks (pre-intervention vs. post-intervention) and interventions (active stimulation vs. sham stimulation). To acquire eye tracking data, eye movements were recorded with a sampling rate of ~50 Hz and an accuracy of approximately 0.5°-1.1° via the infrared-based Tobii eye tracker built into the head-mounted display (HMD). While the software developmental kit (SDK) SRanipal version 1.3.1.1 (HTC Corporation, Taoyuan, Taiwan) procured access to the eye tracking raw data within Unity, the Tobii XR SDK version 1.16.36.0 (Tobii Technology, Stockholm, Sweden) allowed to track the participant’s momentary gaze on specified virtual objects within the VSR. Specifically, it was tracked when and for how long the participants looked at the canvas as well as on 3D objects that were implemented as distracting events (during DP). Offline analyses were run in Matlab 2021b (MathWorks Inc., Natick, MA, United States). To statistically compare gaze locations for each block and intervention, three parameters were extracted (Selaskowski et al., 2023a): Time looking at canvas (as a measure of task focus), time looking at distractors (as a measure of focus on specific distractors) and time of gaze wandering (i.e., that time amount the participants neither looked at a distractor nor at the canvas). Moreover, based on these three derived parameters, a composite distractibility score was calculated by dividing the sum of the time of looking at distractors (in %) and time of gaze-wandering (in %) by the time of looking at canvas (in %), with higher values indicating a higher level of distraction.

#### 2.7.6 Actigraphy recording and analyses

Actigraphy analyses focused on differences in head position shifts and head rotations between blocks (pre-intervention vs. post-intervention) and interventions (sham stimulation vs. active stimulation). The two actigraphy parameters were inferred from the built-in positional tracking of the Vive system by means of which the HMDs momentary positions and rotations during the experiment were each recorded with a ~ 90 Hz sampling rate in three-dimensional Euclidean space coordinates. For offline analyses, actigraphy data was first down-sampled to 10 Hz. Next, the Euclidean distance between each sample point (three-dimensional position or rotation vector) and its preceding sample point was specifically calculated for the HMD position and HMD rotation data. Finally, to statistically compare the amount of head position shifts and rotations between conditions, the mean Euclidean distance in respect to head position shifts and head rotations was derived for each block and intervention.

### 2.8 Data exclusion

Twelve participants had to be excluded from the overall analyses: three because they refrained from the study after the diagnostic appointment or first measurement date; four because of technical difficulties (on at least one experimental day, EEG measurements were aborted or key presses were not recorded), four because the CPT in these subjects accidentally had a different number of pseudo-targets, and one because there were large outliers in CPT performance. Hence, 15 participants (4 female, *M_age_* = 32.53, *SD* = 11.07) remained for analyses. Two datasets did not contain eye tracking data, hence only 13 datasets remained for these analyses. Considering a power analysis for a within-between interaction, a sample size of *n* = 16 would be required to establish reliable results with an effect size of *η*^2^ = 0.14 and a power of 0.80. The effect sizes of our study exceeded these with *η*^2^ = 0.23 for the EEG alpha power interaction effect, thereby determining the *post-hoc* power to 97.5% for this model (see section 3.4). Therefore, the obtained sample should be sufficient to detect potential tACS effects.

### 2.9 Statistical analyses

For statistical analyses with Matlab 2021b (MathWorks Inc., Natick, MA, United States), the following outcome variables were included: omission error rate, commission error rate, and RTV for the CPT analysis; hyperactivity, inattention, and impulsivity ratings for the subjective ADHD symptom evaluation; mean alpha power for the wavelet analysis; composite distractibility score, gaze time on canvas, gaze time on distractors and gaze-wandering time for eye tracking analysis; and head movement and rotation for actigraphy analyses. For each main dependent variable, a two-way repeated measures ANOVA with the two within-factors “Block” (pre-intervention vs. post-intervention) and “Intervention” (active stimulation vs. sham stimulation) was conducted, with an α-level of 0.05. In case of a significant interaction, we followed up this interaction via *post-hoc t*-tests (sham pre vs. sham post; active pre vs. active post; sham pre vs. active pre; sham post vs. active post). In order to correct for multiple comparison by Bonferroni correction, only those *p* < 0.0125 (α-level of 0.05/4 *post-hoc* tests) were considered as statistically significant.

## 3 Results

### 3.1 Sample characteristics

Results of the eligibility assessment and clinical characterization are reported in Table 1. Out of the 15 participants analyzed (4 female, *M_age_* = 32.53, *SD* = 11.07), 14 participants (93.3%) were found to have a combined ADHD presentation and one participant (6.7%) had a predominantly inattentive presentation. None of our participants were assigned to the impulsive–hyperactive subtype. An ADHD diagnosis had been evident since childhood in 12 participants (80%). Six patients received ADHD-medication. Moreover, five patients took selective serotonin reuptake inhibitors or selective serotonin-noradrenalin-reuptake-inhibitors for the treatment of depression or anxiety. Most participants had a higher education entrance qualification (73.3%). The most common current comorbidities found were anxiety disorders (53.3%) and affective disorders (46.7%). According to the depression-anxiety-scales (DASS-21; Nilges and Essau, 2015), participants revealed, on average, only low scores for symptoms of depression (*M* = 12.73; *SD* = 2.91), anxiety (*M* = 12.13; *SD* = 3.11) and stress (*M* = 15.00; *SD* = 3.70).

**Table 1:** Demographic and clinical characteristics of the sample.

Total sample (*n*)	15
Female [*n* (*%*)]	4 (26.67)
Age [*M* (*SD*)]	32.53 (11.07)

Interview data		
**IDA-R**		**Maximum scores**
**ADHD presentations [*n* (%)]**		
Combined type	14 (93.33)	
Predominantly hyperactive–impulsive type	0	
Predominantly inattentive type	1 (6.67)	
**ADHD scores [*M* (*SD*)]**		
Total	35.60 (6.20)	54
Inattention	19.80 (3.41)	27
Hyperactivity	9.13 (2.88)	15
Impulsivity	6.67 (3.09)	12

Mini-DIPS^*^		
	**Current diagnosis (*n*)**	**Previous diagnosis (*n*)**
Affective disorder	6	2
Anxiety disorder	5	0
Somatoform disorder	1	0
Sleep disorder	2	1

Questionnaire data:		
**ADHS-SB**	***M*** (***SD***)	**Maximum scores**
Total	45.67 (9.54)	54
Inattention	24.67 (4.59)	27
Hyperactivity	11.87 (3.42)	15
Impulsivity	9.13 (2.90)	12
**WHOQOL**		**Maximum scores**
Total	61.08 (13.44)	100
Physical health	59.66 (18.12)	100
Psychological health	49.72 (19.70)	100
Social relationships	62.22 (16.33)	100
Environment	72.71 (14.87)	100
**DASS-21**		**Maximum scores**
Total	13.29 (2.26)	21
Depression	12.73 (2.91)	21
Anxiety	12.13 (3.11)	21
Stress	15.00 (3.70)	21

Results of the eligibility assessment and clinical characterization of the sample. ^*^Only comorbidities with > 0 occurrences are reported. Maximum scores for IDA-R and ADHD-SB depict sum scores, while for WHQOL and DASS mean scores.

Most frequently reported tACS side effects, according to the questionnaire about tACS side effects (Brunoni et al., 2011), were fatigue (*n* = 12 per condition, 80%), whereby only two participants (13.4%) in the active stimulation condition associated fatigue symptoms with tACS, but rather linking it to the experiment duration. In addition, difficulties in concentration and headaches were reported (for detailed results, see Supplementary material 2). This implies that during the experiment, participants experienced some discomfort, but no one aborted the experiment and no serious adverse events occurred. Checking for blinding, analyses revealed that for active stimulation 9 participants (60%) detected the condition correctly.

### 3.2 Behavioral performance

**Figure 2:**
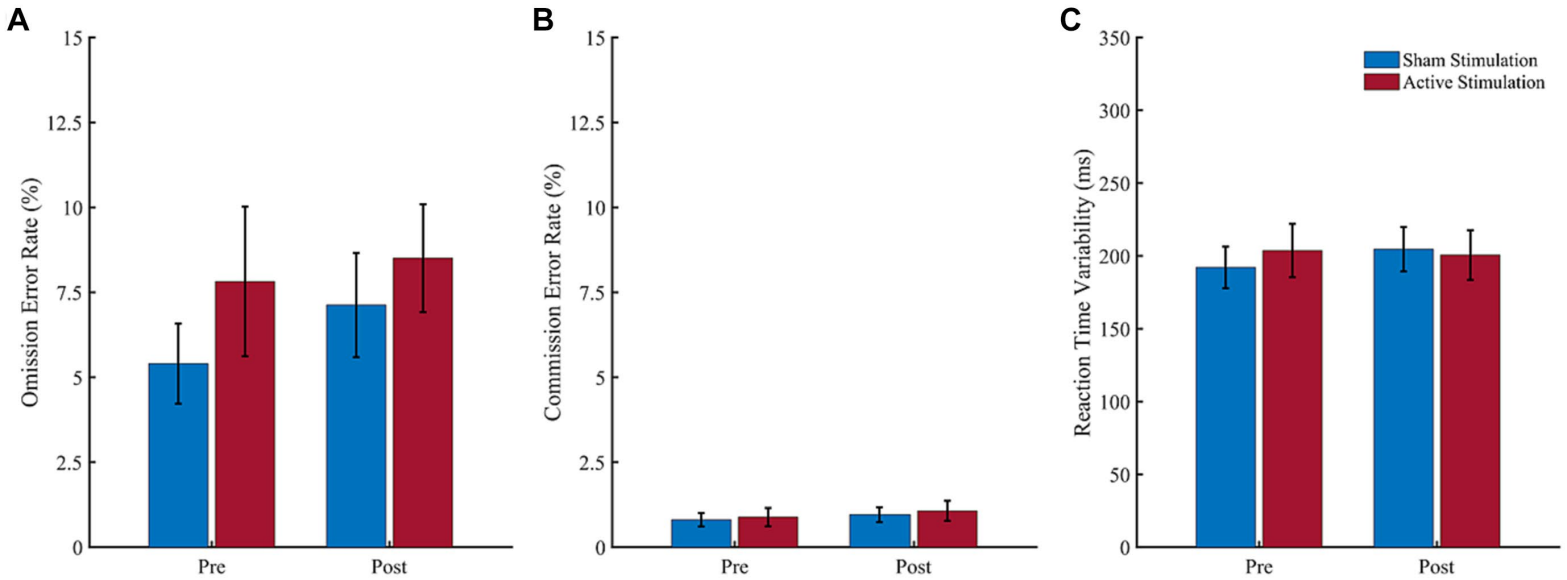
Results of the CPT. Values depict means for the **(A)** omission error rate, **(B)** commission error rate and **(C)** reaction time variability before (pre) and after (post) sham stimulation (blue bars) and active stimulation (red bars). Error bars represent the standard error of the mean.

Results of the CPT analyses are shown in Figure 2. Regarding omission error rate (Figure 2A), the ANOVA revealed neither a significant main effect of “Block” (*F* (1, 14) = 0.96, *p* = 0.347, *η*_p_^2^ = 0.06), nor a main effect of “Intervention” (*F* (1, 14) = 1.48, *p* = 0.244, *η*_p_^2^ = 0.10) and no interaction effect (*F* (1, 14) = 0.22, *p* = 0.647, *η*_p_^2^ = 0.02). Also, for commission error rate (Figure 2B), the ANOVA revealed neither a significant effect of “Block” (*F* (1, 14) = 3.27, *p* = 0.092, *η*_p_^2^ = 0.19), nor a significant effect of “Intervention” (*F* (1, 14) = 0.36, *p* = 0.557, *η*_p_^2^ = 0.03), and no interaction effect (*F* (1, 14) = 0.04, *p* = 0.848, *η*_p_^2^ = 0.00) was found. And finally, the ANOVA for reaction time variability (Figure 2C) yielded neither a significant main effect of “Block” (*F* (1, 14) = 0.33, *p* = 0.577, *η*_p_^2^ = 0.02) or “Intervention” (*F* (1, 14) = 0.14, *p* = 0.712, *η*_p_^2^ = 0.01), nor an interaction effect (*F* (1, 14) = 1.12, *p* = 0.307, *η*_p_^2^ = 0.07).

### 3.3 Subjective ADHD symptom evaluation

**Figure 3:**
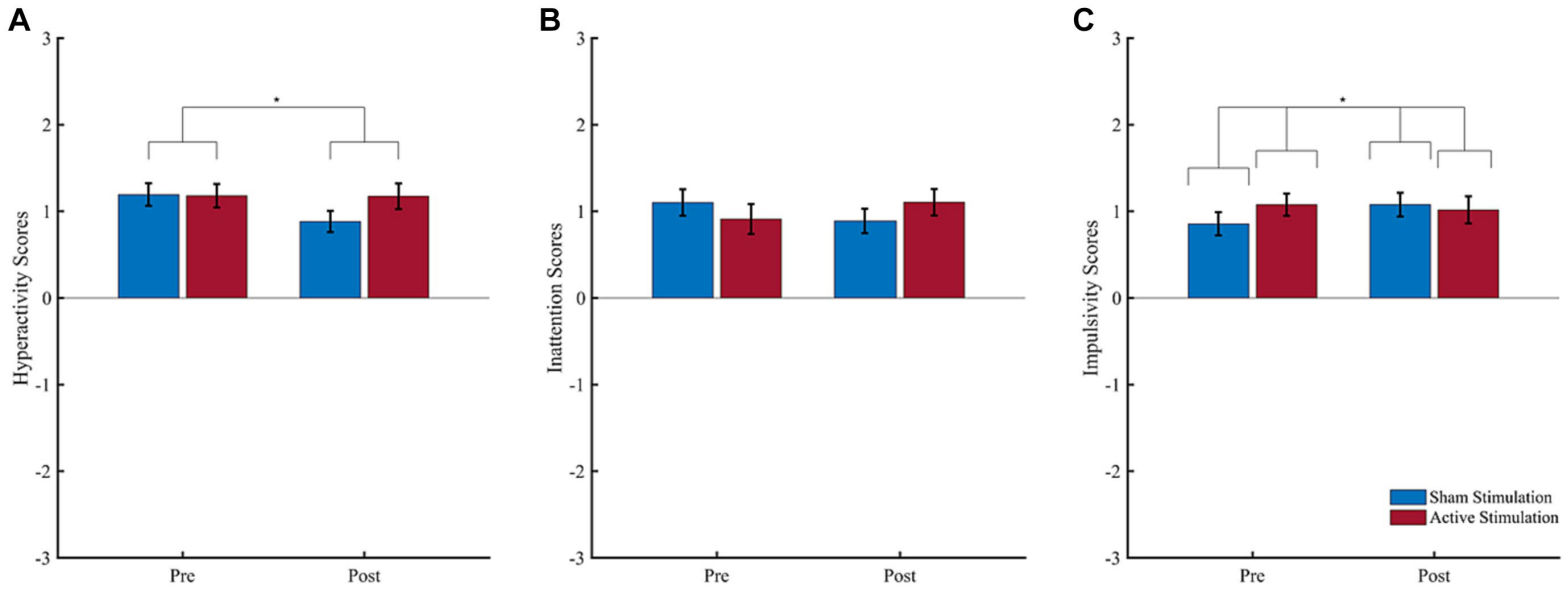
Subjective ratings of core ADHD symptoms. Patient-rated symptoms of **(A)** hyperactivity, **(B)** inattention, and **(C)** impulsivity before (pre) and after (post) intervention. Scores ranged from −3 (strongly disagree) to 3 (strongly agree). Error bars represent the standard error of the mean. ^*^p < 0.05.

Results of the subjective evaluations are shown in Figure 3. For reported hyperactivity (Figure 3A), the ANOVA revealed a significant effect of “Block” (*F* (1, 14) = 5.38, *p* = 0.036, *η*_p_^2^ = 0.28), but no significant effect of “Intervention” (*F* (1, 14) = 2.34, *p* = 0.148, *η*_p_^2^ = 0.14) and no interaction effect (*F* (1, 14) = 2.13, *p* = 0.167, *η*_p_^2^ = 0.13). The significant “Block” effect consisted of higher hyperactivity scores during the pre-intervention (*M* = 1.19; *SD* = 0.45) than post-intervention (*M* = 1.03; *SD* = 0.45) block.

For reported inattention (Figure 3B), in turn, the ANOVA revealed neither a significant effect of “Block” (*F* (1, 14) = 0.03, *p* = 0.862, *η*_p_^2^ = 0.00) nor an effect of “Intervention” (*F* (1, 14) = 0.01, *p* = 0.939, *η*_p_^2^ = 0.00), and no interaction effect (*F* (1, 14) = 3.81, *p* = 0.071, *η*_p_^2^ = 0.21). Finally, regarding reported impulsivity (Figure 3C), the ANOVA revealed no significant main effect of “Block” (*F* (1, 14) = 0.44, *p* = 0.648, *η*_p_^2^ = 0.03), or “Intervention” (*F* (1, 14) = 1.91, *p* = 0.188, *η*_p_^2^ = 0.12), but a significant interaction effect (*F* (1, 14) = 3.40, *p* = 0.048, *η*_p_^2^ = 0.20). Following up this interaction effect, Bonferroni corrected paired *t*-tests neither revealed a significant difference between pre- to post- intervention for active stimulation (*t* (14) = 0.33, *p* = 0.746) nor sham stimulation (*t* (14) = −1.99, *p* = 0.067). All other follow-up *t*-test were non-significant.

### 3.4 Wavelet analysis

**Figure 4:**
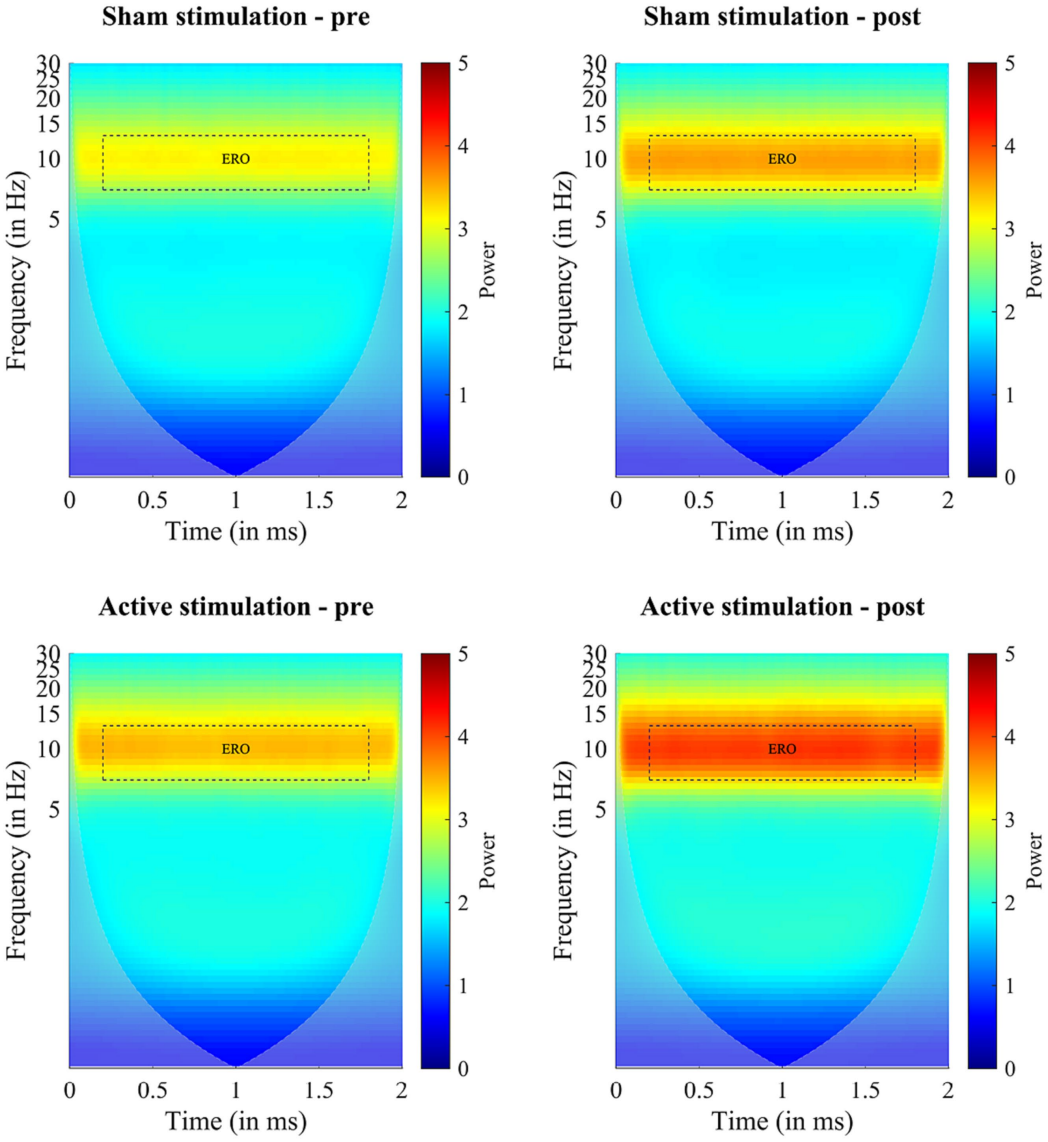
Results of the Wavelet analyses during CPT performance before (pre) and after (post) intervention (sham stimulation vs. active stimulation). Data analyses based on *n* = 15 datasets. ERO = event related potential.

**Figure 5:**
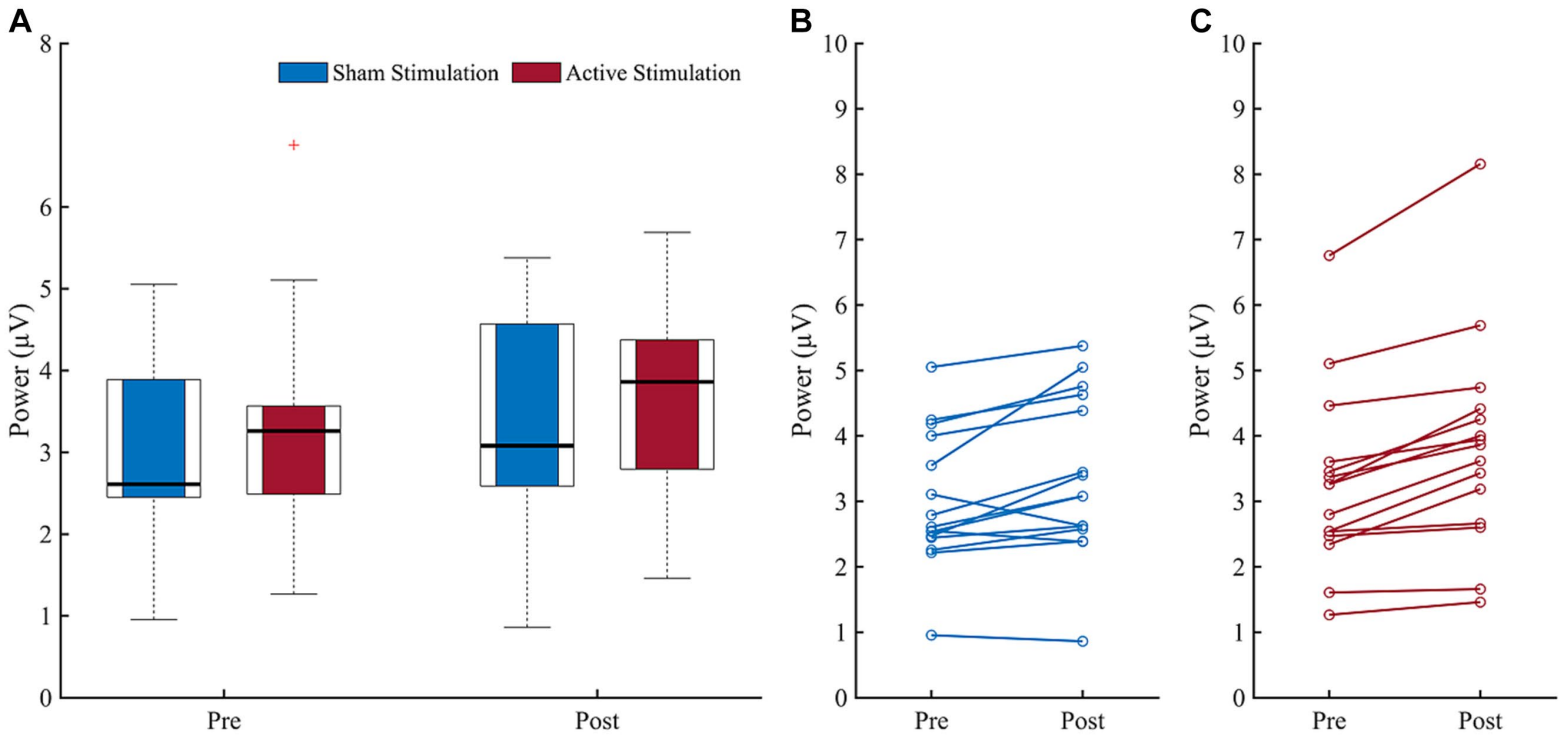
Block comparison (pre vs. post) of individual mean alpha power. **(A)** Boxplots depict mean alpha power before (pre) and after (post) for sham stimulation (blue) and active stimulation (red). **(B)** Pre to post change of individual mean alpha power for sham stimulation and **(C)** for active stimulation. Data analyses based on *n* = 15 datasets. Error bars represent the standard error of the mean.

Before starting the actual experiment, the mean alpha frequency outside VR amounted to *M* = 9.63 (*SD* = 0.69) in the stimulation group and *M* = 9.67 (*SD* = 0.98) in the sham group. Results of the wavelet analysis during CPT are shown in Figure 4, while the individual mean alpha power during CPT before and after both interventions are depicted in Figure 5. The ANOVA on the mean alpha power revealed no significant main effect for “Intervention” (*F* (1, 14) = 0.97, *p* = 0.342, *η*_p_^2^ = 0.06), but a significant main effect of “Block” (*F* (1, 14) = 23.11, *p* < 0.001, *η*_p_^2^ = 0.62), and a trend for an interaction effect (*F* (1, 14) = 4.19, *p* = 0.060, *η*_p_^2^ = 0.23). The block effect resulted from higher amplitude values during the post-intervention block (*M* = 3.61, *SD* = 1.25) compared to the pre-intervention block (*M* = 3.13, *SD* = 0.10). Following up the trend for an interaction exploratively, we see a significant increase from pre- to post-measurements during sham stimulation (*t* (14) = −3.14, *p* = 0.007) and active stimulation (*t* (14) = −5.64, *p* = <0.001), even after Bonferroni correction. All other follow-up *t*-test were non-significant.

Results of the wavelet analyses during the two-minutes resting phases, are, in turn, depicted in the Supplementary material 1. Here, the ANOVA revealed a significant main effect of “Block” (*F* (1, 14) = 9.87, *p* = 0.007, *η*_p_^2^ = 0.41), but no significant effect for “Intervention” (*F* (1, 14) = 3.60, *p* = 0.079, *η*_p_^2^ = 0.20), and only a trend for an interaction effect (*F* (1, 14) = 4.09, *p* = 0.063, *η*_p_^2^ = 0.23). Following up on the trend for an interaction exploratively, after applying the Bonferroni correction, none of the paired *t*-tests yielded statistically significant differences in any of the tests conducted.

### 3.5 Eye tracking

**Figure 6:**
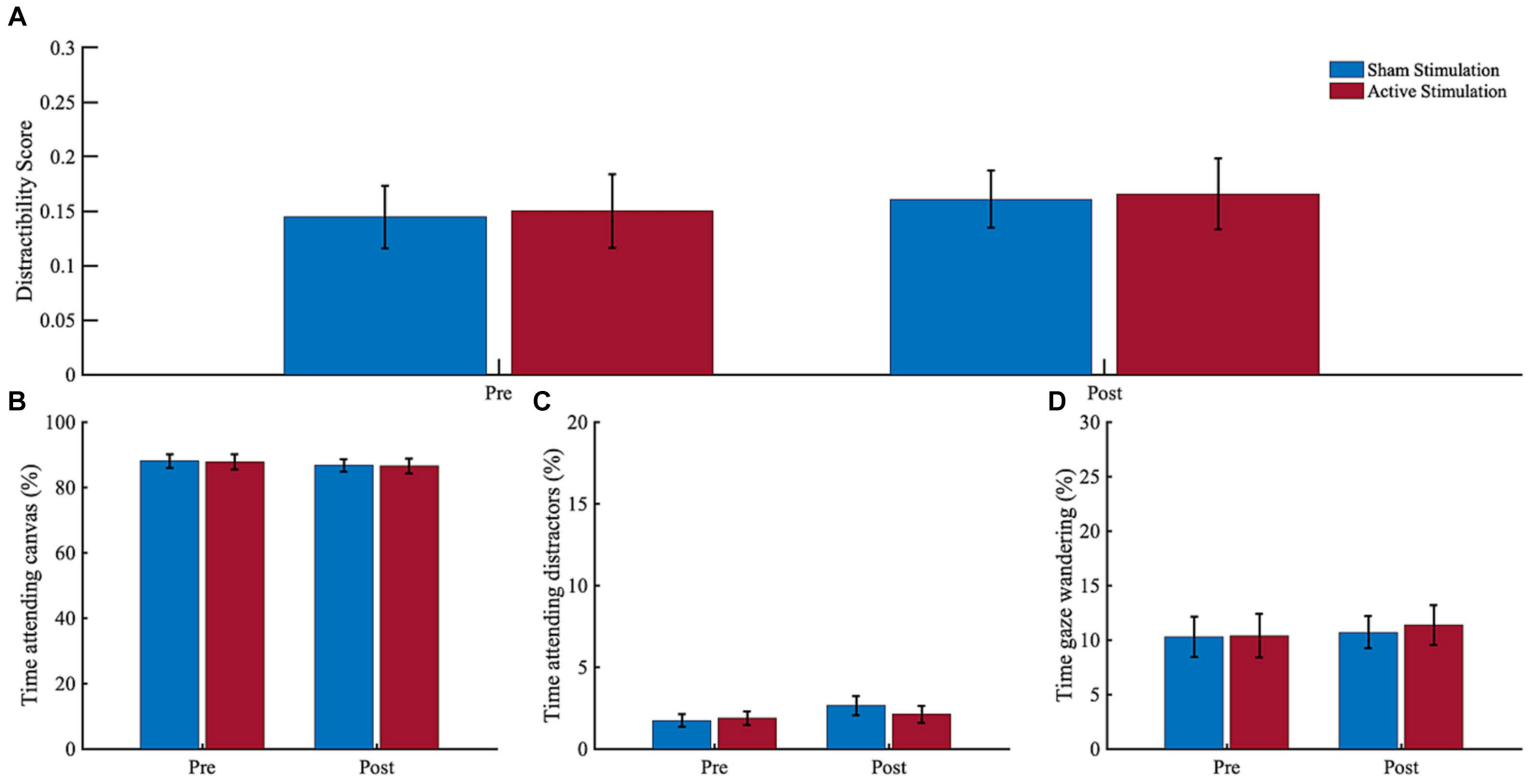
Eye tracking results. **(A)** Distractibility score, **(B)** Time attending canvas, **(C)** Time attending distractors, and **(D)** Time gaze wandering. Dwell time percentages before (pre) and after (post) sham stimulation (blue bars) and active stimulation (red bars). Data analyses based on *n* = 13 datasets. Error bars represent the standard error of the mean.

Results of the eye tracking analyses are depicted in Figure 6. The ANOVA for gaze time on canvas revealed no significant main effect of “Block” (*F* (1, 12) = 2.23, *p* = 0.161, *η*_p_^2^ = 0.16), or “Intervention” (*F* (1, 12) = 0.01, *p* = 0.914, *η*_p_^2^ = 0.00), and no significant interaction (*F* (1, 12) = 0.01, *p* = 0.942, *η*_p_^2^ = 0.00). For the gaze time looking on distractors, in turn, there was a trend for “Block” (*F* (1, 12) = 4.47, *p* = 0.056, *η*_p_^2^ = 0.27), but no effect for “Intervention” (*F* (1, 12) = 0.32, *p* = 0.580, *η*_p_^2^ = 0.03) or the interaction (*F* (1, 12) = 3.21, *p* = 0.098, *η*_p_^2^ = 0.21). The trend effect indicated potentially higher gaze time on distractors during the post-intervention block (*M* = 4.75, *SD* = 3.71) compared to the pre-intervention block (*M* = 3.60, *SD* = 2.61). The ANOVA for gaze wandering revealed no significant main effect of “Block” (*F* (1, 12) = 0.77, *p* = 0.396, *η*_p_^2^ = 0.06), or “Intervention” (*F* (1, 12) = 0.05, *p* = 0.824, *η*_p_^2^ = 0.00), and no significant interaction (*F* (1, 12) = 0.16, *p* = 0.700, *η*_p_^2^ = 0.01).

### 3.6 Actigraphy

**Figure 7:**
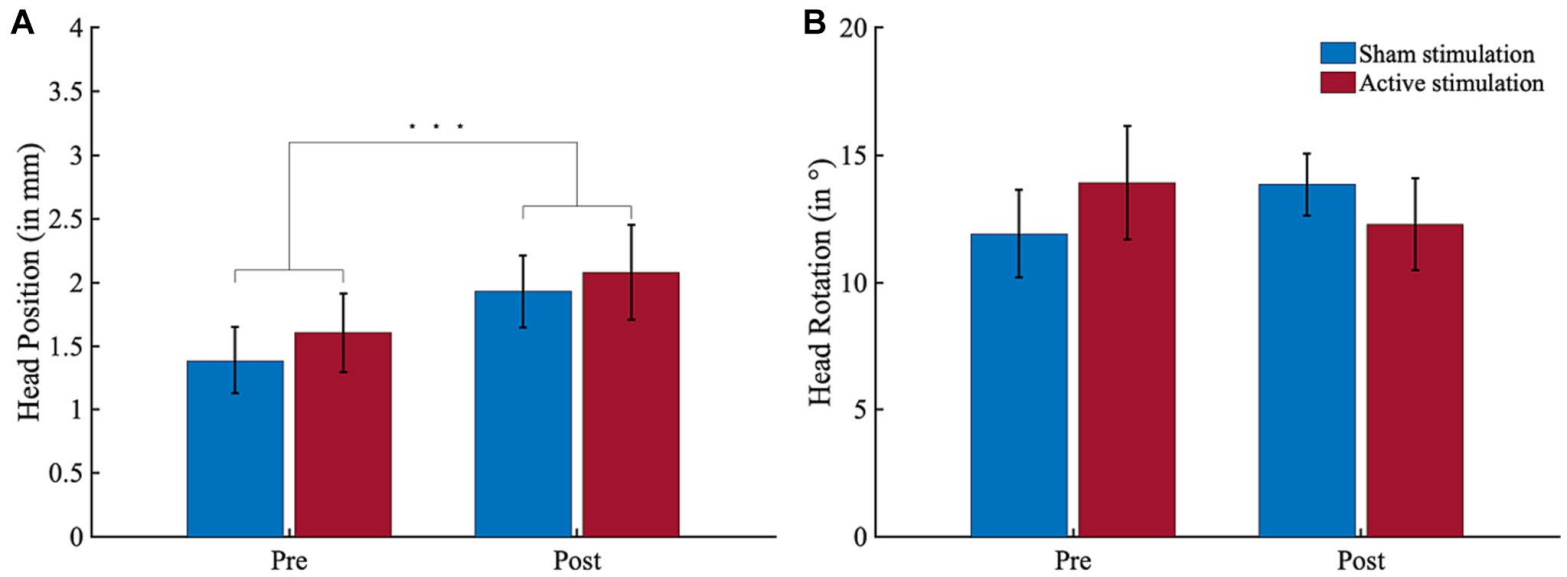
Actigraphy results. **(A)** Head position (in mm per 100 ms) and **(B)** head ration (in ° per 100 ms) shifts in block 1 (pre-intervention) and block 3 (post-intervention). All participants conducted greater head position shifts during block 3 than block 1. Error bars represent the standard error of the mean. ^***^p < 0.001.

Results of the actigraphy analyses are depicted in Figure 7. For head position, there was a significant effect for “Block” (*F* (1, 14) = 18.83, *p* < 0.001, *η*_p_^2^ = 0.57) but neither for “Intervention” (*F* (1, 14) = 0.70, *p* = 0.418, *η*_p_^2^ = 0.05) nor for the interaction (*F* (1, 14) = 0.08, *p* = 0.776, *η*_p_^2^ = 0.01). The block effect resulted from higher head position scores in the post-intervention block (*M* = 4.01, *SD* = 2.26) compared to the pre-intervention block (*M* = 3.00, *SD* = 2.10). For head rotation, there was no significant effect for “Block” (*F* (1, 14) = 0.02, *p* = 0.897, *η*_p_^2^ = 0.00) or “Intervention” (*F* (1, 14) = 0.01, *p* = 911, *η*_p_^2^ = 0.00), and no significant interaction (*F* (1, 14) = 3.61, *p* = 0.078, *η*_p_^2^ = 0.21).

## 4 Discussion

Given the evidence for a decreased EEG alpha power in adult ADHD (Loo et al., 2009; Woltering et al., 2012; Poil et al., 2014; Liu et al., 2016; Deiber et al., 2020), the objective of the current study was to increase the alpha power of adult ADHD patients and to explore possible resulting neurophysiological and/or behavioral changes. Therefore, we carried out a crossover trial, in which a final sample of *n* = 15 adult patients with ADHD underwent both an individual tACS-based alpha stimulation (active stimulation) and a placebo stimulation (sham stimulation) while performing a CPT in a VSR scenario. We examined the mean alpha power at rest (2 min each) and during CPT conductance (18 min each), CPT performances, subjective ADHD symptoms, head movement and rotation, and gaze behavior before and after both interventions.

While alpha power significantly increased from pre- to post-interventions, we were not able to find a significantly stronger increase in alpha power due to active stimulation compared to sham stimulation, neither at rest nor during CPT execution. Although both statistical analyses each yielded a trend for a significant interaction, exploratively assessed trend interactions indicated time differences rather than intervention effects. While the block effect can be attributed to a natural alpha rise in both groups, which is a well-known phenomenon during a prolonged cognitive task as a function of time on task and mental fatigue (Fan et al., 2015; Gharagozlou et al., 2015; Trejo et al., 2015; Benwell et al., 2019), it is not clear why we do not find a significant difference in the participants’ alpha power comparing the application of active and sham stimulation. Nevertheless, since we only expect a small effect of tACS anyway and, in addition, the effect of tACS is quite variable, the small sample size is a constraint in our study. It seems that a larger sample size could have resulted in a significant effect.

In addition, patients with different ADHD presentations seem to show varying levels of alpha power. Most studies suggest a decreased alpha power in patients with ADHD (Loo et al., 2009; Woltering et al., 2012; Poil et al., 2014; Liu et al., 2016; Deiber et al., 2020), but some studies also report an increased alpha power (Koehler et al., 2009; Poil et al., 2014; Deiber et al., 2020), especially for those suffering from hyperactivity/impulsivity (Deiber et al., 2020). Of note, our ADHD sample almost exclusively consisted of patients with the combined ADHD presentation. Hence, almost all our patients also exhibited a level of hyperactivity, which might be associated with a higher and therefore not strongly further increasable alpha power. This indicates that a subgroup of ADHD patients (e.g., a predominantly inattentive sample) associated with a diminished alpha power, might have benefited more from the tACS application. However, since our data seem to show a high variability in the alpha power pre-to-post change (*cf.* Figures 5B,C), further basic research is needed to clarify whether abnormal alpha power is a neuromarker for a specific ADHD subtype, and to what extent subtype-specific neural activity patterns need to be taken more into account in the application of tACS.

Finally, the success of brain stimulation might have been influenced by inter- and intraindividual variability, e.g., by an unfavorable brain state during the application of tACS or by using a non-individualized electrode montage that failed to target the correct source (Bergmann, 2018; von Conta et al., 2021; Kasten and Herrmann, 2022). This could have affected the subsequent aftereffects (the so called “offline effects” that we have investigated) of induced synaptic changes by non-invasive brain stimulation (for details, see, e.g., Vossen et al., 2015). One possible innovative approach to overcome the individual variability would be to use a closed loop system that tracks brain activity during tACS application and adjusts the stimulation accordingly (Zrenner et al., 2016). Since there are only few studies investigating online adaptation of stimulation parameters depending on current brain activity so far, the efficiency and practicability of such closed loop systems needs to be further evaluated (Bergmann et al., 2016; Karabanov et al., 2016; Thut et al., 2017; Stecher et al., 2021).

Regarding behavioral measures, we found no indication for a tACS-induced cognitive improvement for any of our CPT performance, eye tracking, actigraphy or subjective measures. In sum, our tACS application does not appear to have induced any clinically meaningful effect in terms of behavioral changes.

### 4.1 Task related time-on-task effects

Regarding pre-post effects, one interesting finding is that there was a higher gaze time spent on distractors as well as a higher amount of head position movements in the post-intervention block as compared to the pre-intervention block. The latter result is consistent with the results of a virtual classroom study in ADHD children by Mühlberger et al. (2020) as well as with our own VSR study in healthy controls (Wiebe et al., 2022), which both yielded very similar time-on-task head movement effects. Regarding gaze duration on distractors, the outcome agrees with Wiebe et al. (2023), who found that unmedicated ADHD patients spent significantly more time gazing at distracting stimuli while being immersed into the VSR, compared to healthy controls. Interpreting both results, it could be assumed that our participants became increasingly inattentive and/or restless over the duration of the experiment. This, in turn, may suggest that our VSR setup was able to induce the neuropsychologically-desired boredom and monotony in our participants that may provoke inattention, hyperactivity, and impulsivity in adults with ADHD. If this is true, this induction of monotony was, however, insufficiently small, as no pre-post effect was found for any of the CPT performance measures.

Another finding is that in contrast to the pre-post increase of head movements, participants reported to be less hyperactive in the post-intervention block as compared to the pre-intervention block. In other words, while the participants perceived that their motor activity decreased over the course of the experiment, their motor activity increased. One possible explanation for this mismatch between active and experienced movement behavior might be a “positive illusory bias” (i.e., an overestimation of one’s own competence that does not correspond to one’s active performance) that has already been repeatedly reported for ADHD children (Owens et al., 2007; Prevatt et al., 2012; Volz-Sidiropoulou et al., 2016) and recently also for ADHD adults (Butzbach et al., 2021). Another alternative explanation might be habituation. That is, our participants got used to the experimental procedure and virtual surrounding and thereby became less excited over time, what resulted in diminished feeling of restlessness. Likewise, it is also conceivable that head movements might not be a reliable marker of hyperactivity in patients with ADHD. Nevertheless, these diverging outcomes underline the importance of a multimodal assessment when testing the efficacy of tACS or other therapeutic interventions in ADHD, as our data suggests that one cannot rely on subjective data alone.

### 4.2 Limitations and future directions

A limitation of this study is the small final sample size (*n* = 15). Reasons for this included our technically challenging multimodal VR paradigm, which caused some technical difficulties during data acquisition, as well as an impeded ADHD patient access due to the Corona pandemic. Our data suggest that a stimulation effect might have been found with a larger sample. Moreover, a larger sample could indicate the extent to which the specific ADHD presentation might be associated with a significant stimulation effect.

Another aspect to be considered is that, in addition to the studies cited for decreased alpha power (Loo et al., 2009; Woltering et al., 2012; Poil et al., 2014; Liu et al., 2016; Deiber et al., 2020), there is also some evidence for equal (van Dongen-Boomsma et al., 2010) or even increased alpha power (Bresnahan and Barry, 2002; Koehler et al., 2009) in adult ADHD patients compared to healthy individuals. Assuming that the alpha power is increased, the mechanism of action proposed in this study to achieve attentional improvement through alpha amplification might be ineffective, since an already elevated endogenous alpha power cannot be further increased by tACS (Neuling et al., 2013). To account for heterogeneity, future studies might evaluate the alpha power of adult ADHD patients beforehand and allocate them accordingly into groups of low and high alpha power before applying tACS to test its therapeutic effect. Additionally, further work is needed to explore the potential differential effects of tACS on the different ADHD subtypes, thereby contributing to a more detailed understanding of its potential therapeutic applicability. Unfortunately, in the present study it was not possible to conduct such an analysis, as the majority of our participants was diagnosed with the combined ADHD type and only one participant with the predominantly inattentive subtype, thereby precluding a subgroup analysis.

It is also conceivable that other potential ADHD neuromarkers could be considered for tACS. One possibility might be the theta-beta-ratio (TBR), which seems encouraging since TBR differences between children with ADHD and healthy controls appear to exist (Monastra et al., 2001; Snyder and Hall, 2006; Zhang et al., 2017). The prospect of using tACS to correct this ratio would offer a non-invasive therapeutic approach aimed at improving attention and cognitive deficits in the ADHD population. Another promising option would be to enhance the P300 (Prox et al., 2007; Itagaki et al., 2011; Marquardt et al., 2018) by the application of tACS. Some studies already aimed for this goal (Dallmer-Zerbe et al., 2020; Kannen et al., 2022). A recent study by Boetzel et al. (2023) accomplished to increase the P300 amplitude in healthy controls but revealed no dependent effect on behavioral performance parameters yet.

Finally, to our knowledge, this study is one of the first attempting to increase the alpha power of adult ADHD patients using tACS. In addition, we combined the application of tACS with a multimodal VR assessment, creating a functional setup in which various measurement techniques (EEG, eye tracking, actigraphy, behavioral performance, subjective measures) are used to investigate a potential stimulation effect in psychophysiological, behavioral, and subjective domains. In fact, there are many different possibilities to apply tACS by changing stimulation parameters (e.g., stimulation intensity), electrode positions, electrode size, or stimulation frequency, which is why further studies will need to be undertaken.

## 5 Conclusion

In conclusion, our study provides no evidence that tACS can increase the alpha power in adult ADHD patients. With a larger sample, however, there might have been a significant difference, since the analyses revealed large effect sizes. Since alpha power in adult ADHD has not yet been investigated in depth and since there are still many conceivable parameter settings for the application of tACS, more research is needed to clarify whether alpha power enhancement via tACS could be advantageous as a possible therapeutic intervention for ADHD. Overall, we have succeeded in creating a multimodal experimental design including multiple measures (subjective, behavioral, electrophysiological, actigraphy, and eyetracking) to test the potential effects of tACS on adult ADHD and our research has raised numerous questions that require further investigation.

## Data availability statement

The raw data supporting the conclusions of this article will be made available by the authors, without undue reservation.

## Ethics statement

The studies involving humans were approved by Medical ethics committee of the University of Bonn. The studies were conducted in accordance with the local legislation and institutional requirements. Written informed consent for participation in this study was provided by the participants. Written informed consent was obtained from the individual(s) for the publication of any potentially identifiable images or data included in this article.

## Author contributions

KK: Conceptualization, Formal analysis, Investigation, Methodology, Project administration, Visualization, Writing – original draft, Writing – review & editing. JR: Investigation, Writing – review & editing. AF: Investigation, Writing – review & editing. AW: Formal analysis, Writing – review & editing. BS: Formal analysis, Writing – review & editing. LA: Writing – review & editing. BA: Writing – review & editing. SL: Writing – review & editing. CSH: Conceptualization, Methodology, Supervision, Writing – review & editing. AP: Conceptualization, Funding acquisition, Supervision, Writing – review & editing, Methodology. NB: Conceptualization, Formal analysis, Methodology, Supervision, Visualization, Writing – original draft, Writing – review & editing.

## Footnotes

^1^ https://www.redmedical.de

^2^ https://github.com/sccn/labstreaminglayer
