# Supplementary material for: Alpha modulation via transcranial alternating current stimulation in adults with attention-deficit hyperactivity disorder
Source: Front Psychol. 2024 Jan 12;14:1280397. doi: 10.3389/fpsyg.2023.1280397 (PMC10812111; doi:10.3389/fpsyg.2023.1280397)
Supplement: Supplementary file 1 [file Data_Sheet_1.DOCX]

Supplementary Material

Alpha Modulation via Transcranial Alternating Current Stimulation in Adults with Attention-Deficit Hyperactivity Disorder

**Kyra Kannen, Johanna Rasbach, Amin Fantazi, Annika Wiebe, Benjamin Selaskowski, Laura Asché, Behrem Aslan, Silke Lux, Christoph S. Herrmann, Alexandra Philipsen, Niclas Braun***

*** Correspondence: Dr. Niclas Braun**: Niclas.Braun@ukbonn.de

**Supplementary Material 1
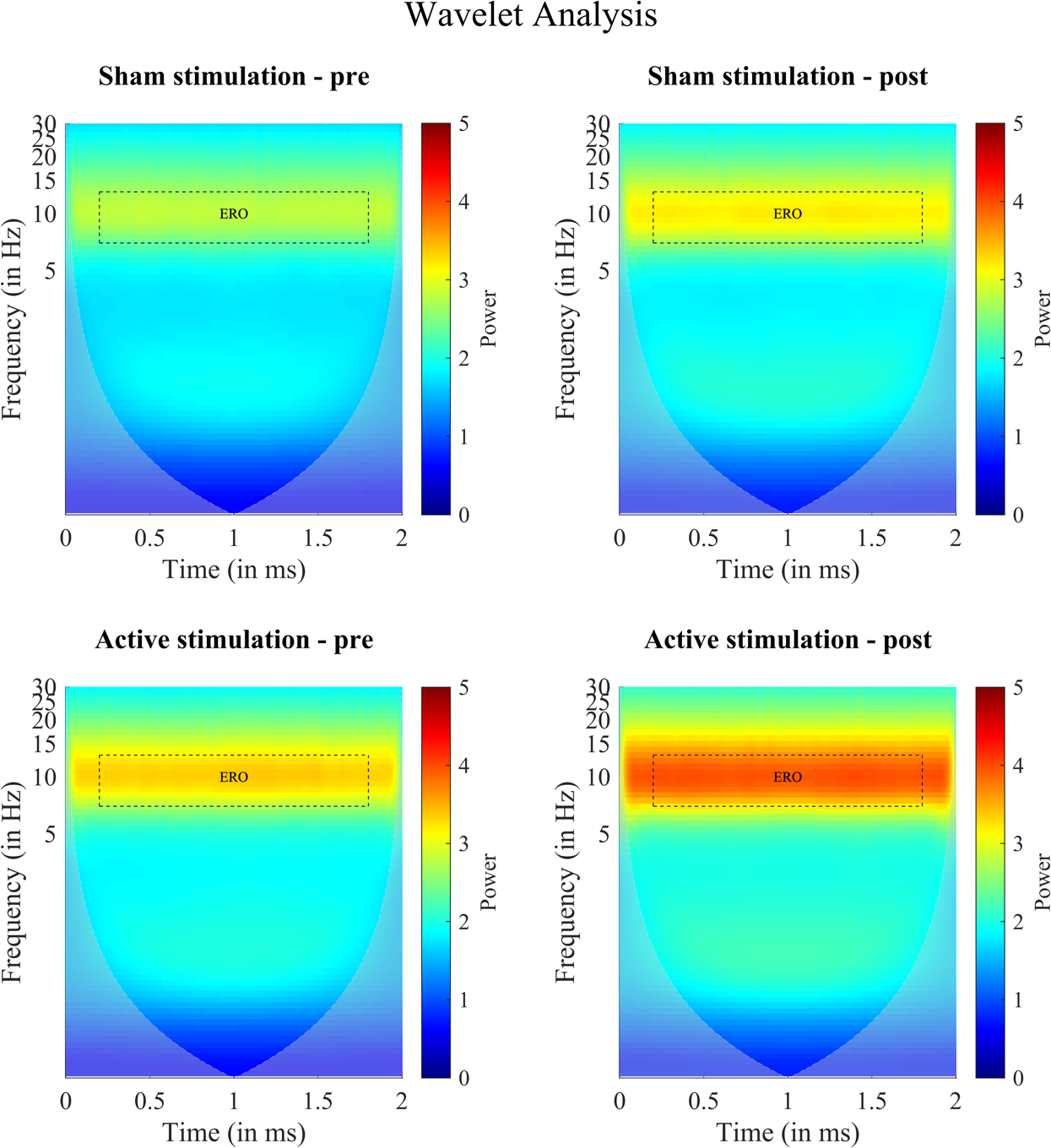
**

**Supplementary Figure 1.** Wavelet analysis of the resting period.

**Supplementary Table 1.** Outcomes of the questionnaire assessing tACS side effects (*N* = 15).

**Supplementary Material 2**

| **Side effect** | **Intervention** | **n (%)** | **Thought to be caused by tACS** |
| --- | --- | --- | --- |
| Headaches | *Active Stimulation* | 4 (26.7%) | 0 |
|  | *Sham Stimulation* | 7 (﻿46.7%) | ﻿1 (6.7%) |
| Cervical pain | *Active Stimulation* | 0 | 0 |
|  | *Sham Stimulation* | ﻿2 (13.3%) | 0 |
| Pain on scalp | *Active Stimulation* | ﻿4 (26.7%) | ﻿1 (6.7%) |
|  | *Sham Stimulation* | ﻿4 (26.7%) | ﻿1 (6.7%) |
| Tingling | *Active Stimulation* | ﻿2 (13.3%) | ﻿1 (6.7%) |
|  | *Sham Stimulation* | ﻿5 (33.4%) | ﻿5 (33.4%) |
| Itching | *Active Stimulation* | 0 | 0 |
|  | *Sham Stimulation* | ﻿2 (13.3%) | 0 |
| Feeling hot | *Active Stimulation* | ﻿4 (26.7%) | ﻿2 (13.3%) |
|  | *Sham Stimulation* | ﻿4 (26.7%) | ﻿2 (13.3%) |
| Fatigue | *Active Stimulation* | ﻿12 (80%) | ﻿2 (13.4) |
|  | *Sham Stimulation* | ﻿12 (80%) | 0 |
| Inattention | *Active Stimulation* | ﻿12 (80%) | ﻿1 (6.7%) |
|  | *Sham Stimulation* | ﻿11 (73.3%) | 0 |
| Mood swings | *Active Stimulation* | ﻿3 (20%) | 0 |
|  | *Sham Stimulation* | ﻿2 (13.3%) | 0 |

|  | **Sham Stimulation** | | **Active Stimulation** | |
| --- | --- | --- | --- | --- |
| **Parameter** | **Pre** | **Post** | **Pre** | **Post** |
| CPT performance |  |  |  |  |
| Omission error rate (%) | 5.40 (4.56) | 7.13 (5.94) | 7.82 (8.51) | 8.50 (6.15) |
| Commission error rate (%) | 0.81 (0.77) | 0.96 (0.86) | 0.88 (1.04) | 1.07 (1.13) |
| RTV (s) | 192.08 (55.15) | 204.59 (59.01) | 203.66 (70.88) | 200.62 (66.15) |
| Frequency Analysis |  |  |  |  |
| Alpha Power CPT | 3.00 (1.04) | 3.38 (1.24) | 3.26 (1.38) | 3.84 (1.63) |
| Alpha Power Rest | 3.02 (0.94) | 3.20 (1.08) | 3.73 (1.45) | 3.77 (1.47) |
| Head Movement |  |  |  |  |
| Position (mm) | 1.39 (1.02) | 1.93 (1.10) | 1.61 (1.19) | 2.08 (1.44) |
| Rotation (°) | 11.92 (6.67) | 13.85 (4.69) | 13.91 (8.62) | 12.29 (7.01) |
| Eyetracking |  |  |  |  |
| Gaze time looking on canvas | 88.00 (7.70) | 86.67 (7.06) | 87.75 (8.27) | 86.53 (8.10) |
| Gaze time looking on distractors | 1.73 (1.40) | 2.64 (2.11) | 1.87 (1.53) | 2.11 (1.88) |
| Gaze indicating mindwandering | 10.27 (6.72) | 10.69 (5.38) | 10.38 (7.26) | 11.37 (6.57) |

**Supplementary Table 2.** Descriptive statistic for all main variables. Outcomes indicated as *M* (*SD*).
